# Outcomes of switching to dasatinib after imatinib-related low-grade adverse events in patients with chronic myeloid leukemia in chronic phase: the DASPERSE study

**DOI:** 10.1007/s00277-018-3295-8

**Published:** 2018-03-20

**Authors:** Dong-Wook Kim, Susanne Saussele, Loretta A. Williams, Hesham Mohamed, Yuanxin Rong, Teresa Zyczynski, Javier Pinilla-Ibarz, Elisabetta Abruzzese

**Affiliations:** 1 Seoul St. Mary’s Hospital, Leukemia Research Institute, Catholic Hematology Hospital, The Catholic University of Korea, 222 Banpodaero Seochogu, Seoul, South Korea; 2 III. Med. Klinik, Medizinische Fakultät Mannheim, Universität Heidelberg, Mannheim, Germany; 3 MD Anderson Cancer Center, University of Texas, Houston, TX USA; 4 Bristol-Myers Squibb, Princeton, NJ USA; 5 H. Lee Moffitt Cancer Center and Research Institute, Tampa, FL USA; 6 Ospedale S. Eugenio, Tor Vergata University, Rome, Italy

**Keywords:** Tyrosine kinase, Chronic myeloid leukemia, Adverse effects, Quality of life

## Abstract

Chronic, low-grade adverse events are common in patients with chronic myeloid leukemia who are treated with imatinib. These events may decrease patient quality of life and adherence, and may ultimately contribute to a suboptimal response. Alternative, second-generation tyrosine kinase inhibitors, such as dasatinib, are available with the potential to reduce adverse events, improve tolerability, and support long-term treatment goals. We present the final, primary analysis of DASPERSE/CA180-400 (NCT01660906), an open-label, multicenter, phase IV study designed to determine whether chronic, low-grade nonhematologic adverse events in imatinib-treated patients improve after switching to dasatinib, without affecting efficacy. Of the 121 chronic, grade 1/2, imatinib-related adverse events identified at baseline in 39 patients, 77% resolved or improved within 3 months after switching to dasatinib. Dasatinib maintained a consistent safety profile; headache (33%), pleural effusion (26%), fatigue (23%), and rash (23%) were the most common treatment-related adverse events after the switch. Patients either maintained (56%) or improved (44%) their molecular response on dasatinib. Patients who switched to dasatinib also experienced improved patient-reported symptom burden from baseline as assessed by the MD Anderson Symptom Inventory for chronic myeloid leukemia (on a 1–10 scale, mean change in disease-specific score was − 2.24 and core symptom severity score was − 1.06). Overall, the efficacy and quality of life/symptom burden improved in many patients, despite the onset of dasatinib-related adverse events in most patients. This suggests that imatinib-treated patients with chronic, low-grade adverse events could benefit from switching to treatment with dasatinib.

**Electronic supplementary material:**

The online version of this article (10.1007/s00277-018-3295-8) contains supplementary material, which is available to authorized users.

## Introduction

Patients diagnosed with chronic myeloid leukemia (CML) in chronic phase (CP) and treated with imatinib often experience chronic, low-grade adverse events (AEs) that may negatively impact their quality of life and their ability to remain on long-term therapy [1–5]. Results from the Imatinib Long-Term Side Effects study reported that more than half of patients with CML who were being treated with imatinib have at least one nonserious AE after a median treatment period of 5.8 years, and that nonserious AEs increased over time, from 34.3% at 6 years to 52.6% at 8 years [4].

Persistent AEs can decrease a patient’s quality of life [2, 4], with the impact on daily life potentially being greater in both younger patients (18–39 years) and women [2]. Patients with CML and chronic, imatinib-related AEs have been found to have significantly lower rates of medication adherence [6], which could result in poor therapeutic outcomes, such as suboptimal responses [7, 8].

Dasatinib is approved for first-line treatment of CML-CP and second-line treatment of CML in patients who are resistant to or intolerant of imatinib [9, 10]. Each tyrosine kinase inhibitor (TKI) has its own unique structure and interactions with BCR-ABL1, dasatinib being 325-fold more potent than imatinib at inhibiting BCR-ABL1 in vitro [11]. Differences in TKI structure and binding can also lead to distinct safety profiles for each drug [9, 10, 12]; for example, pleural effusion occurs with higher incidence in patients treated with dasatinib versus imatinib, and facial edema, muscle spasms, myalgia, nausea, and vomiting are reported more frequently with imatinib versus dasatinib [13, 14]. Among the BCR-ABL1 TKIs, pulmonary arterial hypertension (PAH) has been mainly associated with dasatinib, although it is rare and often reversible upon dose reduction/interruption [9, 10].

Assessment of imatinib-intolerant patients who were treated with second-line dasatinib demonstrated that there is a low incidence of nonhematologic cross-intolerance [15]. Only 4% of patients who discontinued imatinib due to grade 3/4 nonhematologic AEs experienced these same AEs when receiving dasatinib. This suggests that dasatinib is a suitable option for patients with CML who are imatinib intolerant. The DASPERSE (CA180-400) study enrolled patients with CML-CP who had achieved an optimal response while treated with imatinib but were experiencing persistent, low-grade AEs. The objective of the study was to evaluate whether after switching to dasatinib these patients had resolution of their imatinib-related toxicities while maintaining or improving their clinical response.

## Methods

### Patients and study design

DASPERSE (CA180-400/NCT01660906) was a phase IV, open-label, multicenter study. Patients with CML-CP who had been treated with imatinib for a minimum of 3 months were enrolled. Inclusion criteria required patients to have at least one current imatinib-related grade 1/2 nonhematologic AE persisting for a minimum of 2 months or recurring at least three times over the previous 12 months, despite best supportive care. To be considered for the study, patients with CML-CP must have achieved an optimal response by 2009 European LeukemiaNet recommendations [16].

Enrolled patients were treated with 100 mg of dasatinib once a day for 12 months, or until disease progression, treatment failure, unacceptable toxicity, or withdrawal of consent. Dose interruptions/reductions were used to treat grade ≥ 2 dasatinib-emergent AEs until AEs resolved or recovered to grade 1. After 12 months on study, patients could continue treatment off study with dasatinib, or another BCR-ABL1 TKI for CML-CP, as prescribed by the treating physician. This trial was approved by all institutional review boards and ethics committees. All patients gave written informed consent before enrollment, in accordance with the Declaration of Helsinki.

The primary endpoint of the study was to determine the frequency of reduction or resolution of grade 1/2, imatinib-related, chronic nonhematologic AEs within 3 months after switching to dasatinib. Additional endpoints included determining the frequency of dasatinib-related AEs, changes in patient-reported symptom burden, the proportion of patients with a grade 1/2 resolution or grade 2 reduction of at least one nonhematologic, imatinib-related AE within 3 months of initiating dasatinib without an increase in any other grade 1/2 imatinib-related AE, and the rate of molecular response after initiating dasatinib.

### Evaluations

All evaluations occurred at baseline and at scheduled times throughout the study; safety and laboratory assessments occurred as scheduled and when deemed clinically necessary by a physician (Online Resource Table S1). AEs were assessed according to the Common Terminology Criteria for Adverse Events from the National Cancer Institute version 4.0. Patient assessment of symptom burden was determined based on the MD Anderson Symptom Inventory for CML (MDASI-CML) [17], work impairment on the Work Productivity and Activity Impairment (WPAI) questionnaire [18], and quality of life on the European Organisation for Research and Treatment of Cancer quality of life (EORTC QLQ-C30) questionnaire [19].

Efficacy was assessed through quantitative reverse transcription polymerase chain reaction (qRT-PCR) on the International Scale (IS). Assessments were performed in a centralized laboratory, and results were shared with the investigator site within 2–3 weeks. Molecular response was categorized into *BCR-ABL1* (IS) transcript levels of ≤ 0.0032, 0.0032 to ≤ 0.1, 0.1 to ≤ 1, 1 to ≤ 10, and > 10%. Major molecular response (MMR) is defined as *BCR-ABL1* (IS) transcripts ≤ 0.1% and MR^4.5^ as *BCR-ABL1* (IS) transcripts ≤ 0.0032% [20].

### Statistical analysis

All patients who had received at least one dose of dasatinib were considered for analysis. Statistical analyses for safety, efficacy, and patient symptom burden are described in the Online Supplementary Methods [21, 22].

## Results

### Patient population

A total of 39 patients with CML-CP and prior imatinib treatment were enrolled between December 7, 2012 and October 1, 2015. The patient population was well balanced for sex, with 54% of patients being male (Table 1). The overall median age was 57 years (range 23–81 years), and 31% of patients were older than 65 years of age. Median time from diagnosis of CML-CP was 51 months (range 4–215 months) prior to enrollment, and the median duration of imatinib treatment was 51 months (range 3–160 months). At baseline, 49% of the patients were receiving a dose of imatinib below the recommended 400 mg/d. Most patients (*n* = 32; 82%) had at least one preexisting medical condition noted in their medical history, and 41% had a cardiovascular-related morbidity. The highest proportion of patients had a best baseline molecular response of MMR (*n* = 20; 51%), followed by MR^4.5^ (*n* = 10; 26%), *BCR-ABL1* 0.1–1% (*n* = 5; 13%), *BCR-ABL1* 1–10% (*n* = 3; 8%), and *BCR-ABL1* > 10% (*n* = 1; 3%).

**Table 1:** Patient baseline characteristics

	Enrolled patients (*N* = 39)
Patient characteristic	
Median age, years (range)	57 (23.0–81.0)
< 65 years, *n* (%)	27 (69.2)
≥ 65 years, *n* (%)	12 (30.8)
Sex, *n* (%)	
Male	21 (53.8)
Female	18 (46.2)
Median time since CML-CP diagnosis, months (range)	51.3 (3.9–214.6)
Median duration of imatinib treatment, months (range)	51.2 (3.1–160.4)
Imatinib dose at baseline, *n* (%)	
< 400 mg	19 (48.7)
400 mg	20 (51.3)
Baseline molecular response to imatinib, *n* (%)	
*BCR-ABL1* > 10%	1 (2.6)
*BCR-ABL1* 1–10%	3 (7.7)
*BCR-ABL1* 0.1–1%	5 (12.8)
*BCR-ABL1* ≤ 0.1% (MMR)	20 (51.3)
*BCR-ABL1* ≤ 0.0032% (MR^4.5^)	10 (25.6)

### Change in imatinib-related AEs on dasatinib

**Fig. 1:**
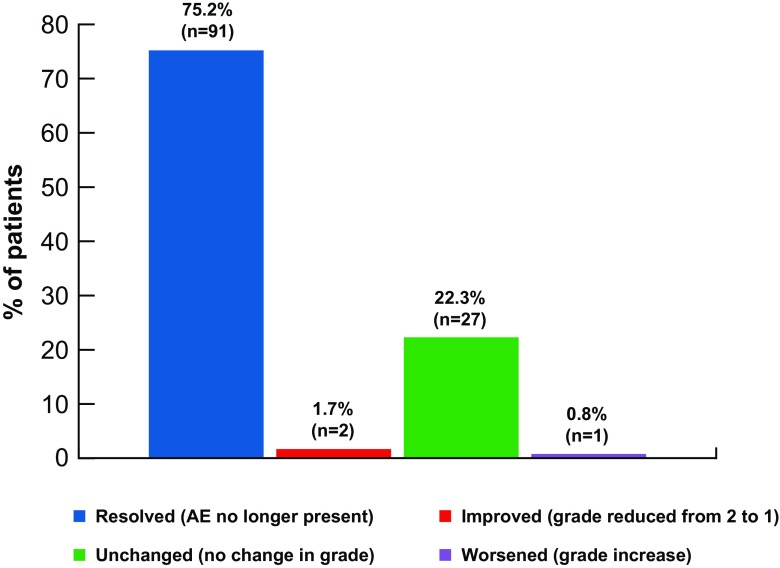
The status of imatinib-related AEs within 3 months after patients had switched to dasatinib. Resolved AEs are those that are no longer present, improved AEs reduced from grade 2 to grade 1, unchanged AEs did not improve or worsen, and worsened AEs had a grade increase

The primary endpoint of the DASPERSE study was to determine the frequency at which chronic, grade 1/2, nonhematologic, imatinib-related AEs either resolved or improved within 3 months of switching to dasatinib. A total of 121 imatinib-related AEs were identified at baseline; 75% of these AEs resolved and 2% improved within 3 months of initiating dasatinib (Fig. 1). The most common imatinib-related AE was muscle spasms (*n* = 22) (Table 2); all but one instance of muscle spasms improved within 3 months after switching to dasatinib. Facial edema (*n* = 10) and rash (*n* = 10) were the next most common AEs at baseline; all but one case of facial edema and two cases of rash improved within 3 months after the dasatinib switch. Age did not seem to have an impact on these results, as 83% and 87% of the most frequent imatinib-related AEs improved in patients aged < 65 and ≥ 65 years, respectively. Improvement of at least one imatinib-related AE without a worsening of any others was reported in 34 (87%) patients with similar results in patients < 65 years (89%) and ≥ 65 years (83%). A ≥ 70% chance of improvement after switching to dasatinib was observed with eight AEs (muscle spasms, periorbital edema, pain, rash, diarrhea, nausea, fatigue, and dry skin) (Online Resource Fig. S1). Overall, only one patient experienced a worsening of an AE reported at baseline (fatigue), from grade 1 to grade 2, which occurred on the first day of the study. The remaining four patients had AEs that were unchanged after switching to dasatinib.

**Table 2:** Status of all baseline chronic, grade 1/2 imatinib-related AEs 3 months after switching to dasatinib

AEs	Imatinib-related AEs, *n* (%) (*n* = 121)			
	Baseline, *n* (%)	After 3 months on dasatinib, *n* (percent of baseline)		
		Resolved	Improved^a^	Unchanged
Musculoskeletal and connective tissue disorders	39 (32.2)	33 (84.6)	0	6 (15.4)
Muscle spasms	22 (18.2)	21 (95.5)	0	1 (4.5)
Myalgia	8 (6.6)	6 (75.0)	0	2 (25.0)
Arthralgia	6 (5.0)	4 (66.7)	0	2 (33.3)
Back pain	1 (0.8)	1 (100)	0	0
Muscular weakness	1 (0.8)	1 (100)	0	0
Pain in extremity	1 (0.8)	0	0	1 (100)
General disorders and administration site conditions	22 (18.2)	17 (77.3)	1 (4.5)	3 (13.6)
Face edema	10 (8.3)	9 (90.0)	0	1 (10.0)
Fatigue	3 (2.5)	0	0	2 (66.7)
Asthenia	2 (1.7)	1 (50.0)	1 (50.0)	0
Generalized edema	2 (1.7)	2 (100)	0	0
Edema	2 (1.7)	2 (100)	0	0
Peripheral edema	2 (1.7)	2 (100)	0	0
Pain	1 (0.8)	1 (100)	0	0
Gastrointestinal disorders	17 (14.0)	12 (70.6)	0	5 (29.4)
Diarrhea	7 (5.8)	5 (71.4)	0	2 (28.6)
Nausea	5 (4.1)	4 (80.0)	0	1 (20.0)
Abdominal pain	1 (0.8)	1 (100)	0	0
Ascites	1 (0.8)	1 (100)	0	0
Cheilitis	1 (0.8)	1 (100)	0	0
Stomatitis	1 (0.8)	0	0	1 (100)
Vomiting	1 (0.8)	0	0	1 (100)
Skin and subcutaneous tissue disorders	17 (14.0)	12 (70.6)	1 (5.9)	4 (23.5)
Rash	10 (8.3)	7 (70.0)	1 (10.0)	2 (20.0)
Skin fragility	3 (2.5)	3 (100)	0	0
Dry skin	2 (1.7)	1 (50.0)	0	1 (50.0)
Alopecia	1 (0.8)	0	0	1 (100)
Erythema	1 (0.8)	1 (100)	0	0
Eye disorders	16 (13.2)	13 (81.3)	0	3 (18.8)
Periorbital edema	6 (5.0)	3 (50.0)	0	3 (50.0)
Eyelid edema	3 (2.5)	3 (100)	0	0
Conjunctival hemorrhage	2 (1.7)	2 (100)	0	0
Lacrimation increased	2 (1.7)	2 (100)	0	0
Eye edema	1 (0.8)	1 (100)	0	0
Eye pain	1 (0.8)	1 (100)	0	0
Visual acuity reduced	1 (0.8)	1 (100)	0	0
Nervous system disorders	4 (3.3)	1 (25.0)	0	3 (75.0)
Headache	2 (1.7)	1 (50.0)	0	1 (50.0)
Dizziness	1 (0.8)	0	0	1 (100)
Paraesthesia	1 (0.8)	0	0	1 (100)
Investigations	2 (1.7)	0	0	2 (100)
Increased blood creatinine	1 (0.8)	0	0	1 (100)
Increased weight	1 (0.8)	0	0	1 (100)
Cardiac disorders	1 (0.8)	1 (100)	0	0
Palpitations	1 (0.8)	1 (100)	0	0
Infections/infestations	1 (0.8)	1 (100)	0	0
Cellulitis	1 (0.8)	1 (100)	0	0
Renal/urinary disorders	1 (0.8)	0	0	1 (100)
Nephropathy	1 (0.8)	0	0	1 (100)
Respiratory, thoracic, and mediastinal disorders	1 (0.8)	1 (100)	0	0
Dyspnea	1 (0.8)	1 (100)	0	0

^a^AE improved from grade 2 to grade 1

### Dasatinib-related AEs

As expected, a total of 34 (87%) patients experienced a dasatinib-related AE while on study treatment. Dasatinib-related AEs were consistent with previous reports of first- and second-line dasatinib treatment [13, 23]. The majority of AEs were grade 1 or 2 in severity, reported in 23 of 34 (68%) patients, and no patients died on study. The frequency of dasatinib-related AEs that occurred in ≥ 5% of patients are listed in Table 3. The most frequent dasatinib-related AEs (any grade) occurring in > 20% of total patients were headache (33%), pleural effusion (26%), fatigue (23%), rash (23%), diarrhea (21%), and dyspnea (21%).

**Table 3:** Frequency of dasatinib-related AEs occurring in ≥ 5% of patients

AE	Patients^a^ with an event, *n* (percent of total patients)		
	All grades	Grade 1/2 (*n* = 23)	Grade 3/4 (*n* = 11)
Nonhematologic AEs			
Headache	13 (33.3)	12 (30.8)	1 (2.6)
Pleural effusion	10 (25.6)	10 (25.6)	0
Fatigue	9 (23.1)	8 (20.5)	1 (2.6)
Rash	9 (23.1)	9 (23.1)	0
Diarrhea	8 (20.5)	6 (15.4)	2 (5.1)
Dyspnea	8 (20.5)	7 (17.9)	1 (2.6)
Nausea	6 (15.4)	6 (15.4)	0
Dizziness	4 (10.3)	4 (10.3)	0
Arthralgia	3 (7.7)	3 (7.7)	0
Pericardial effusion	3 (7.7)	3 (7.7)	0
Pruritus	3 (7.7)	3 (7.7)	0
Facial edema	2 (5.1)	2 (5.1)	0
General health deterioration	2 (5.1)	2 (5.1)	0
Pain in extremity	2 (5.1)	1 (2.6)	1 (2.6)
Paresthesia	2 (5.1)	2 (5.1)	0
Pulmonary hypertension	2 (5.1)	2 (5.1)	0
Pyrexia	2 (5.1)	2 (5.1)	0
Hematologic/laboratory AEs			
Anemia	4 (10.3)	1 (2.6)	3 (7.7)
Increased ALT	2 (5.1)	2 (5.1)	0
Increased blood creatinine	2 (5.1)	0	2 (5.1)

*ALT* alanine aminotransferase

^a^Individual patients can fall into more than one AE category

Grade 3, nonhematologic, dasatinib-related AEs included headache, vomiting, fatigue, dyspnea, pulmonary edema, pain in extremity (each occurring in one patient), and diarrhea (two patients). Dasatinib-related grade 3 AEs from laboratory investigations were anemia and increased blood creatinine in two patients each and increased lipids in one patient. The only grade 4 events were anemia, thrombocytopenia, and hyperuricemia, which occurred in one patient each.

Serious AEs were reported in 11 (28%) patients, with four of these documented as related to dasatinib treatment (pleural effusion, PAH, pulmonary edema, and pyrexia). Other serious AEs, unrelated to treatment, consisted of upper respiratory tract infection, anal fissure, appendicitis, rib fracture, pleural effusion, and pneumothorax (one patient each) and intervertebral disk protrusion, pneumonia, and hemorrhoids (two patients each).

AEs that are of special interest in dasatinib-treated patients include pleural effusion, PAH, and cardiovascular-related events. No patient experienced a drug-related case of grade 3/4 pleural effusion, PAH, pulmonary hypertension (PH), or cardiac disorders while taking dasatinib. Ten of 12 total cases of pleural effusion were dasatinib related: three (8%) patients with grade 1 and seven (18%) patients with grade 2 events. The other two cases of pleural effusion were not considered treatment related. One case of grade 1 PAH, confirmed by right heart catheterization, was documented at the end of the study. Prior to confirmation of PAH, the dasatinib dose was initially reduced and then discontinued after PAH was confirmed, because PAH was determined to be dasatinib related. The patient was then treated with diuretics (i.e., hydrochlorothiazide), which began resolving the PAH. There were also two cases of grade 1 PH identified on the basis of the echocardiogram performed at the end of study. These cases were considered class I PH or PAH according to the revised World Health Organization classification of PH, despite lack of confirmatory right heart catheterization. Both of these patients underwent post-study dose interruptions/reductions of dasatinib, were still displaying symptoms associated with PH (i.e., dyspnea or pleural effusion), and were eventually switched to radotinib, which appeared to improve symptoms. Grade 1/2 cardiac disorders (e.g., pericardial effusion, cardiomegaly, palpitations, sinus tachycardia, and tachycardia) occurred in six (15%) patients.

### Dose modifications

Dasatinib was administered at an average median daily dose of 98 mg (range 25–100 mg), with a median exposure of 12 months (range 1–13 months). There were 23 (59%) patients who underwent a dose interruption once they switched to dasatinib, two patients (5%) with three interruptions, and 21 (54%) with four or more interruptions. Most interruptions were due to a nonhematologic toxicity (19 of 23). Of the 15 (38%) patients who underwent a dose reduction, 13 (33%) had a single dose reduction to 80 mg/d and two (5%) patients had two dose reductions to 80 mg/d and then to 50 mg/d. Two thirds of the dose reductions occurred after a dose interruption, and 87% (13 of 15) of patients were able to continue treatment on a reduced dose.

Three patients discontinued dasatinib due to drug-related AEs; two of these patients discontinued within the first month of initiating dasatinib treatment. One patient had a combination of grade 3 headache, vomiting, fatigue, and diarrhea on the first day of dasatinib treatment. Symptoms resolved after treatment was interrupted. The patient returned to dasatinib treatment at a reduced dose of 50 mg/d, then discontinued dasatinib the following day due to reappearance of drug-related AEs, including grade 2 sinus tachycardia, fatigue, and nausea, and grade 3 headache. The general condition of the patient improved a week after discontinuing dasatinib. Another patient had grade 1 extremity pain and facial edema, grade 2 headache, grade 3 dyspnea, and an increase in nausea from grade 1 to grade 2 on the first day on dasatinib. Grade 2 pleural effusion was then identified within the first month of dasatinib treatment; dasatinib was discontinued and pleural effusion was treated, followed by resolution of all symptoms. The third patient who discontinued dasatinib had grade 2 increased blood creatinine levels on the first day of treatment, which increased further upon dasatinib initiation. Levels were temporarily regulated with dose interruptions and a dose reduction to 80 mg/d, but, 100 days after beginning dasatinib treatment, increased blood creatinine levels progressed from grade 2 to grade 3. The patient’s blood creatinine decreased to grade 2 upon discontinuation of dasatinib.

### Efficacy

**Fig. 2:**
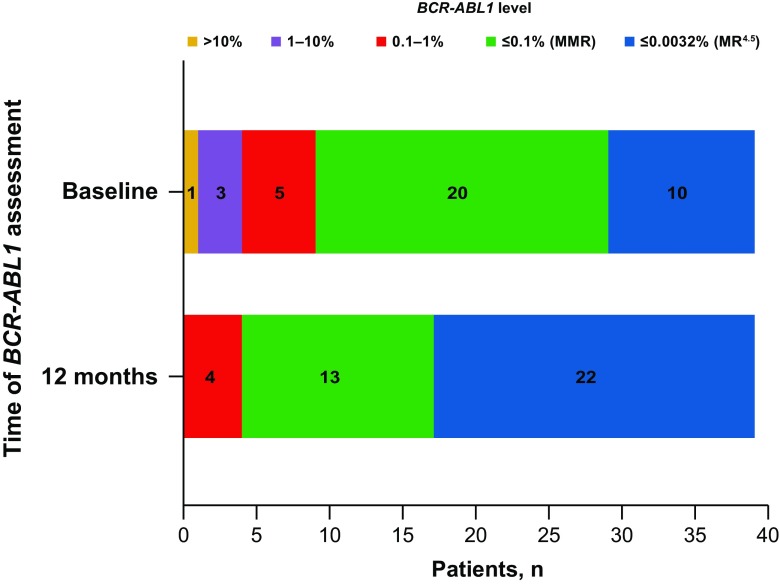
Molecular response of patients at baseline and at 12 months. The number of patients with each molecular response level at baseline and at 12 months is listed by color: MR^4.5^ (blue), MMR (green), *BCR-ABL1* 0.1–≤ 1.0% (red), *BCR-ABL1* 1–10% (purple), and *BCR-ABL1* > 10% (yellow). All patients either maintained or improved their response, and no patients ended the study with *BCR-ABL1* > 1%

Twenty (51%) patients achieved MMR and 10 (26%) achieved MR^4.5^ as their best baseline response on imatinib (Fig. 2). Overall, 17 (44%) patients ended the study with a better response rate than at baseline and no patient had a worsened response, regardless of whether patients completed the study.

Twelve months after switching to dasatinib, the percentage of patients who achieved MR^4.5^ more than doubled from baseline to 56% of patients. At enrollment, there were four patients with a response of > 1% *BCR-ABL1* transcripts (IS); however, all of these patients had improved to a response of *BCR-ABL1* ≤ 1% (IS) at 12 months after switching to dasatinib (Fig. 2). Four (10%) patients had a response rate below MMR at the end of the study, which was *BCR-ABL1* (IS) 0.1 to ≤ 1%.

### Patient-reported outcomes

The impact of symptoms on the daily lives of patients was assessed through two scoring methods, the MDASI-CML survey and EORTC QLQ-C30, to determine changes in symptom severity/interference and patient quality of life, respectively.

**Fig. 3:**
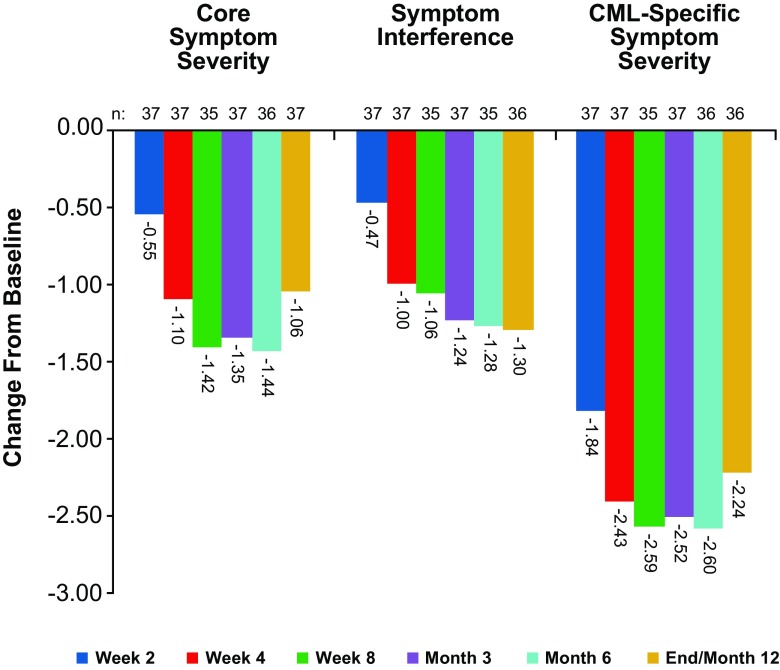
Symptom burden in patients at baseline; weeks 2, 4, and 8; and months 3, 6, and 12 via MD Anderson Symptom Inventory (MDASI) and MDASI for chronic myeloid leukemia (MDASI-CML) scores. The mean change in patient score at each time point is shown, with a negative value indicating an improvement in the symptom being assessed

Improvements in core symptom severity (the first 13 symptom items of the MDASI-CML) and symptom interference were observed as early as 2 weeks after switching to dasatinib (Fig. 3). Symptom severity and interference decreased further at 4 weeks, and improvements were maintained throughout the course of the study. Mean core symptom severity improved from a score of 3.85 at baseline to 2.9 at the end of the study, and symptom interference scores improved from 3.91 to 2.64, respectively. Additional symptoms specific to CML and its treatment (diarrhea, swelling, rash/skin change, muscle soreness/cramping, ease of bruising or bleeding, malaise, and headache) also were assessed with the MDASI-CML [17] survey and showed the same pattern of improvement. CML-specific symptom severity scores improved from a mean of 4.81 at baseline to 2.68 at 12 months. Clinically important differences in MDASI scores (i.e., minimal important difference [MID]) at the end of the study were observed for core symptoms, overall symptoms, CML-specific symptoms, and the five most severe symptoms. Changes from baseline assessment were − 1.06, − 1.46, − 2.24, and − 1.43, respectively.

Patient-reported quality of life was also assessed via the EORTC QLQ-C30 questionnaire, and improvements in both emotional and social functioning were reported. Overall, the global health status of patients was improved through the 12 months after the dasatinib switch. Reductions in activity impairment, as assessed by the WPAI questionnaire, also improved and were sustained throughout the course of dasatinib treatment.

## Discussion

The DASPERSE study aimed to determine whether chronic, grade 1/2 AEs that occurred as a result of imatinib treatment would resolve or improve if patients with CML were switched to the second-generation TKI dasatinib. The initial planned analysis was for 75 patients; however, due to positive results at the interim analysis (*n* = 25; 65% resolution or improvement in chronic, low-grade AEs) and slow enrollment, a decision to terminate the enrollment process early was made. In this final analysis of 39 patients, the majority (93 [77%]) of the 121 imatinib-related AEs documented at baseline were resolved or improved within 3 months after switching to dasatinib. These results align with a patient-centric focus to CML therapy, where treatment is tailored to each individual patient, minimizing chronic AEs and promoting positive therapeutic outcomes. Individualized care is important for patients with CML, as they can experience a nearly normal life span with well-managed TKI therapy [24]. This management includes proper medication adherence; however, chronic AEs can have a negative impact on adherence, resulting in reduced efficacy and poor long-term outcomes [6–8, 25]. Therefore, focusing on individual patient care and minimizing chronic AEs can both improve a patient’s quality of life and help achieve therapeutic milestones and long-term survival.

Baseline assessment identified the most common imatinib-related AEs as facial edema, muscle spasms, and rash, which together comprised 35% of all documented imatinib-related AEs. Muscle spasms, eye/periorbital edema, and rash each had a ≥ 70% chance of resolution or improvement within 3 months of switching to dasatinib. Most patients (34 of 39 [87%]) experienced improvement of an imatinib-related AE, without a worsening of other imatinib-related toxicities, while on dasatinib, thus supporting the established tolerable safety profile and no cross-intolerance of dasatinib [13, 23].

AEs that did emerge once patients switched to dasatinib were consistent with those previously reported in dasatinib clinical trials [13, 23]. Although no new safety signals were reported, one limitation of this study is the small patient number, which could lead to differences in the incidence of AEs compared with previous reports. Pleural effusion occurred in 10 (26%) patients who switched to dasatinib, all of which were grade 1/2 cases. Another clinically important, but rare, event that has been associated with dasatinib treatment is PAH [9, 10]. One case of PAH was confirmed in DASPERSE, which led to discontinuation; however, as echocardiograms were not regularly performed beyond assessments at baseline and study closure, it is unknown if this patient had elevated pulmonary artery pressure earlier in the study, prior to the PAH diagnosis. Important to note, the definition of intolerance in DASPERSE differed from that used in the second-line dasatinib after imatinib intolerance/resistance clinical trial (CA180-034) [13, 23]. In DASPERSE, intolerance included grade ≥ 2 nonhematologic toxicities, whereas in CA180-034 intolerance required grade ≥ 3 toxicities that led to discontinuation. The more stringent definition in DASPERSE, including grade 2 AEs, helped align the focus of the study on patient centricity to encourage provision of the most tolerable treatment for each individual.

Data from DASPERSE suggest that switching TKIs can help resolve chronic AEs, decrease patient symptom burden, and improve patient quality of life. A switch may also improve patient adherence to treatment because certain symptoms, such as nausea and muscle cramps, have been shown to be associated with intentional nonadherence to treatment [26]. Even with these improvements; however, it is critical to ensure that appropriate molecular responses are maintained after changing treatment. The level and sustainability of molecular response is becoming a critical efficacy marker in clinical trials enrolling patients on long-term treatment who wish to attempt TKI discontinuation [27–29]. Previous findings in the CA180-034 study, where 41% of patients received imatinib for at least 3 years, demonstrated improved efficacy and tolerability with second-line dasatinib after imatinib intolerance/resistance [23, 30]. Importantly, patients enrolled in DASPERSE were either able to maintain or improve their baseline molecular response level, regardless of whether patients completed the study. The percentage of patients with MR^4.5^ increased from 26% (10 of 39) at baseline to 56% (22 of 39) of patients at 12 months.

Safety and molecular response outcomes were also investigated with the second-generation TKI nilotinib in the phase II ENRICH (Exploring Nilotinib to Reduce Imatinib Related Chronic Adverse Events) study [31]. DASPERSE and ENRICH had similar study designs to evaluate outcomes when patients with low-grade imatinib-related AEs were switched to a second-generation TKI. Resolution or improvement of imatinib-related AEs and a sustained molecular response were observed in both DASPERSE and ENRICH [31]. In the ENRICH study, 16% of imatinib-related AEs resolved and then reappeared within 12 months; no resolved AEs reemerged in DASPERSE. Treatment-related AEs were reported in 87% of dasatinib-treated patients in DASPERSE and 85% of nilotinib-treated patients in ENRICH. Nilotinib was discontinued in 15% of patients due to AEs and dasatinib in 8% of patients due to AEs, one of these being due to pleural effusion in each study. Dose modifications were employed in both DASPERSE and ENRICH to allow patients to continue, or temporarily interrupt, treatment without a complete cessation of therapy. Overall, these results demonstrate that second-generation TKIs, as alternative options to imatinib therapy, provide clinicians with the opportunity to tailor treatment to each patient’s unique characteristics.

Despite the emergence of some dasatinib-related AEs after switching from imatinib, improvements in quality of life outcomes through MDASI-CML symptom scoring assessment were observed in the DASPERSE study. Quality of life assessments were also improved in the ENRICH study after patients switched from imatinib to nilotinib. The decreased symptom interference and severity scores, which were observed as early as 2 weeks after switching to dasatinib, indicate improvements in patient quality of life, which could ultimately impact long-term outcomes [7, 25]. Clinically important differences in MDASI scores were calculated based on MID values calculated as half a standard deviation of the value determined at patient screening [21]. MID was observed for core symptoms, overall symptoms, CML-specific symptoms, and the five most severe symptoms when comparing MDASI scores at screening to those 12 months after patients were switched to dasatinib. The majority of dasatinib-related AEs were low grade, and only three patients discontinued dasatinib therapy while on the study. This supports the improved quality of life documented by the variety of assessments performed in DASPERSE.

## Conclusions

The DASPERSE study demonstrates that patients with CML can switch from first-line imatinib treatment to dasatinib and experience improvements in chronic, low-grade AEs soon after changing therapies. Improvement in the quality of life of patients and decreased symptom burden occurred early and were sustained during treatment. The treatment-emergent AEs that occurred while patients were taking dasatinib were consistent with previous reports, largely low grade, not chronic, and rarely led to discontinuation of therapy. Moreover, molecular responses were consistently improved or maintained. Patients experiencing chronic grade 1/2 AEs with imatinib treatment can experience various benefits, including resolution/improvement of chronic AEs, a high, sustained molecular response, and an overall improved quality of life by switching to treatment with dasatinib.

## Electronic supplementary material

ESM 1 (DOCX 107 kb)

## References

[1] Druker BJ, Guilhot F, O'Brien SG, Gathmann I, Kantarjian H, Gattermann N, Deininger MW, Silver RT, Goldman JM, Stone RM, Cervantes F, Hochhaus A, Powell BL, Gabrilove JL, Rousselot P, Reiffers J, Cornelissen JJ, Hughes T, Agis H, Fischer T, Verhoef G, Shepherd J, Saglio G, Gratwohl A, Nielsen JL, Radich JP, Simonsson B, Taylor K, Baccarani M, So C, Letvak L, Larson RA, IRIS Investigators (2006). Five-year follow-up of patients receiving imatinib for chronic myeloid leukemia. N Engl J Med.

[2] Efficace F, Baccarani M, Breccia M, Alimena G, Rosti G, Cottone F, Deliliers GL, Baratè C, Rossi AR, Fioritoni G, Luciano L, Turri D, Martino B, Di Raimondo F, Dabusti M, Bergamaschi M, Leoni P, Simula MP, Levato L, Ulisciani S, Veneri D, Sica S, Rambaldi A, Vignetti M, Mandelli F, GIMEMA (2011). Health-related quality of life in chronic myeloid leukemia patients receiving long-term therapy with imatinib compared with the general population. Blood.

[3] Efficace F, Baccarani M, Breccia M, Cottone F, Alimena G, Deliliers GL, Baratè C, Specchia G, Di Lorenzo R, Luciano L, Turri D, Martino B, Stagno F, Dabusti M, Bergamaschi M, Leoni P, Simula MP, Levato L, Fava C, Veneri D, Sica S, Rambaldi A, Rosti G, Vignetti M, Mandelli F (2013). Chronic fatigue is the most important factor limiting health-related quality of life of chronic myeloid leukemia patients treated with imatinib. Leukemia.

[4] Gambacorti-Passerini C, Antolini L, Mahon FX, Guilhot F, Deininger M, Fava C, Nagler A, Della Casa CM, Morra E, Abruzzese E, D’Emilio A, Stagno F, le Coutre P, Hurtado-Monroy R, Santini V, Martino B, Pane F, Piccin A, Giraldo P, Assouline S, Durosinmi MA, Leeksma O, Pogliani EM, Puttini M, Jang E, Reiffers J, Valsecchi MG, Kim DW (2011). Multicenter independent assessment of outcomes in chronic myeloid leukemia patients treated with imatinib. J Natl Cancer Inst.

[5] Steegmann JL, Baccarani M, Breccia M, Casado LF, García-Gutiérrez V, Hochhaus A, Kim DW, Kim TD, Khoury HJ, Le Coutre P, Mayer J, Milojkovic D, Porkka K, Rea D, Rosti G, Saussele S, Hehlmann R, Clark RE (2016). European LeukemiaNet recommendations for the management and avoidance of adverse events of treatment in chronic myeloid leukaemia. Leukemia.

[6] Marin D, Bazeos A, Mahon FX, Eliasson L, Milojkovic D, Bua M, Apperley JF, Szydlo R, Desai R, Kozlowski K, Paliompeis C, Latham V, Foroni L, Molimard M, Reid A, Rezvani K, de Lavallade H, Guallar C, Goldman J, Khorashad JS (2010). Adherence is the critical factor for achieving molecular responses in patients with chronic myeloid leukemia who achieve complete cytogenetic responses on imatinib. J Clin Oncol.

[7] Ibrahim AR, Eliasson L, Apperley JF, Milojkovic D, Bua M, Szydlo R, Mahon FX, Kozlowski K, Paliompeis C, Foroni L, Khorashad JS, Bazeos A, Molimard M, Reid A, Rezvani K, Gerrard G, Goldman J, Marin D (2011). Poor adherence is the main reason for loss of CCyR and imatinib failure for chronic myeloid leukemia patients on long-term therapy. Blood.

[8] Noens L, van Lierde MA, De Bock R, Verhoef G, Zachée P, Berneman Z, Martiat P, Mineur P, Van Eygen K, MacDonald K, De Geest S, Albrecht T, Abraham I (2009). Prevalence, determinants, and outcomes of nonadherence to imatinib therapy in patients with chronic myeloid leukemia: the ADAGIO study. Blood.

[9] National Comprehensive Cancer Network (2017) NCCN Clinical Practice Guidelines in Oncology (NCCN Guidelines) for Chronic Myeloid Leukemia v.2.2017. NCCN website. [cited 2017 Mar 31]. Available from: https://www.nccn.org/professionals/physician_gls/pdf/cml.pdf

[10] Baccarani M, Deininger MW, Rosti G, Hochhaus A, Soverini S, Apperley JF, Cervantes F, Clark RE, Cortes JE, Guilhot F, Hjorth-Hansen H, Hughes TP, Kantarjian HM, Kim DW, Larson RA, Lipton JH, Mahon FX, Martinelli G, Mayer J, Muller MC, Niederwieser D, Pane F, Radich JP, Rousselot P, Saglio G, Saussele S, Schiffer C, Silver R, Simonsson B, Steegmann JL, Goldman JM, Hehlmann R (2013). European LeukemiaNet recommendations for the management of chronic myeloid leukemia: 2013. Blood.

[11] O’Hare T, Walters DK, Stoffregen EP, Jia T, Manley PW, Mestan J, Cowan-Jacob SW, Lee FY, Heinrich MC, Deininger MW, Druker BJ (2005). In vitro activity of Bcr-Abl inhibitors AMN107 and BMS-354825 against clinically relevant imatinib-resistant Abl kinase domain mutants. Cancer Res.

[12] Abruzzese E, Breccia M, Latagliata R (2014). Second-generation tyrosine kinase inhibitors in first-line treatment of chronic myeloid leukaemia (CML). BioDrugs.

[13] Cortes JE, Saglio G, Kantarjian HM, Baccarani M, Mayer J, Boqué C, Shah NP, Chuah C, Casanova L, Bradley-Garelik B, Manos G, Hochhaus A (2016). Final 5-year study results of DASISION: the Dasatinib Versus Imatinib Study in Treatment-Naive Chronic Myeloid Leukemia Patients trial. J Clin Oncol.

[14] Jabbour E, Kantarjian HM, Saglio G, Steegmann JL, Shah NP, Boqué C, Chuah C, Pavlovsky C, Mayer J, Cortes J, Baccarani M, Kim DW, Bradley-Garelik MB, Mohamed H, Wildgust M, Hochhaus A (2014). Early response with dasatinib or imatinib in chronic myeloid leukemia: 3-year follow-up from a randomized phase 3 trial (DASISION). Blood.

[15] Khoury HJ, Goldberg SL, Mauro MJ, Stone RM, Deininger MW, Bradley-Garelik MB, Mohamed H, Guilhot F (2016). Cross-intolerance with dasatinib among imatinib-intolerant patients with chronic phase chronic myeloid leukemia. Clin Lymphoma Myeloma Leuk.

[16] Baccarani M, Cortes J, Pane F, Niederwieser D, Saglio G, Apperley J, Cervantes F, Deininger M, Gratwohl A, Guilot F, Hochhaus A, Horowitz M, Hughes T, Kantarjian H, Larson R, Radich J, Simonsson B, Silver RT, Goldman J, Hehlmann R, European LeukemiaNet (2009). Chronic myeloid leukemia: an update of concepts and management recommendations of European LeukemiaNet. J Clin Oncol.

[17] Williams LA, Garcia Gonzalez AG, Ault P, Mendoza TR, Sailors ML, Williams JL, Huang F, Nazha A, Kantarjian HM, Cleeland CS, Cortes JE (2013). Measuring the symptom burden associated with the treatment of chronic myeloid leukemia. Blood.

[18] Reilly MC, Zbrozek AS, Dukes EM (1993). The validity and reproducibility of a work productivity and activity impairment instrument. Pharmacoeconomics.

[19] Aaronson NK, Ahmedzai S, Bergman B, Bullinger M, Cull A, Duez NJ, Filiberti A, Flechtner H, Fleishman SB, de Haes JC (1993). The European Organization for Research and Treatment of Cancer QLQ-C30: a quality-of-life instrument for use in international clinical trials in oncology. J Natl Cancer Inst.

[20] Cross NC, White HE, Müller MC, Saglio G, Hochhaus A (2012). Standardized definitions of molecular response in chronic myeloid leukemia. Leukemia.

[21] Cleeland CS (2009) The M. D. Anderson Symptom Inventory. User Guide. Version 1. University of Texas MD Anderson Cancer Center, Houston, TX. https://www.mdanderson.org/documents/Departments-and-Divisions/Symptom-Research/MDASI_userguide.pdf. Accessed 22 Dec 2016

[22] Norman GR, Sloan JA, Wyrwich KW (2003). Interpretation of changes in health-related quality of life: the remarkable universality of half a standard deviation. Med Care.

[23] Shah NP, Rousselot P, Schiffer C, Rea D, Cortes JE, Milone J, Mohamed H, Healey D, Kantarjian H, Hochhaus A, Saglio G (2016). Dasatinib in imatinib-resistant or -intolerant chronic-phase, chronic myeloid leukemia patients: 7-year follow-up of study CA180-034. Am J Hematol.

[24] Baccarani M, Efficace F, Rosti G (2014). Moving towards patient-centered decision-making in chronic myeloid leukemia: assessment of quality of life and symptom burden. Haematologica.

[25] Ganesan P, Sagar TG, Dubashi B, Rajendranath R, Kannan K, Cyriac S, Nandennavar M (2011). Nonadherence to imatinib adversely affects event free survival in chronic phase chronic myeloid leukemia. Am J Hematol.

[26] Efficace F, Rosti G, Cottone F, Breccia M, Castagnetti F, Iurlo A, Mandelli F, Baccarani M (2014). Profiling chronic myeloid leukemia patients reporting intentional and unintentional non-adherence to lifelong therapy with tyrosine kinase inhibitors. Leuk Res.

[27] CML treatment-free remission trials. https://clinicaltrials.gov/ct2/results?term=treatment+free+remission&type=&rslt=&recr=&age_v=&gndr=&cond=CML&intr=&titles=&outc=&spons=&lead=&id=&state1=&cntry1=&state2=&cntry2=&state3=&cntry3=&locn=&rcv_s=&rcv_e=&lup_s=&lup_e=. Accessed 20 Dec 2016

[28] Hughes TP, Ross DM (2016). Moving treatment-free remission into mainstream clinical practice in CML. Blood.

[29] Mori S, Vagge E, le Coutre P, Abruzzese E, Martino B, Pungolino E, Elena C, Pierri I, Assouline S, D’Emilio A, Gozzini A, Giraldo P, Stagno F, Iurlo A, Luciani M, De Riso G, Redaelli S, Kim DW, Pirola A, Mezzatesta C, Petroccione A, Lodolo D’Oria A, Crivori P, Piazza R, Gambacorti-Passerini C (2015). Age and dPCR can predict relapse in CML patients who discontinued imatinib: the ISAV study. Am J Hematol.

[30] Shah NP, Kantarjian HM, Kim DW, Réa D, Dorlhiac-Llacer PE, Milone JH, Vela-Ojeda J, Silver RT, Khoury HJ, Charbonnier A, Khoroshko N, Paquette RL, Deininger M, Collins RH, Otero I, Hughes T, Bleickardt E, Strauss L, Francis S, Hochhaus A (2008). Intermittent target inhibition with dasatinib 100 mg once daily preserves efficacy and improves tolerability in imatinib-resistant and -intolerant chronic-phase chronic myeloid leukemia. J Clin Oncol.

[31] Cortes JE, Lipton JH, Miller CB, Busque L, Akard LP, Pinilla-Ibarz J, Keir C, Warsi G, Lin FP, Mauro MJ (2016). Evaluating the impact of a switch to nilotinib on imatinib-related chronic low-grade adverse events in patients with CML-CP: the ENRICH study. Clin Lymphoma Myeloma Leuk.

