# Supplementary material for: Outcomes of switching to dasatinib after imatinib-related low-grade adverse events in patients with chronic myeloid leukemia in chronic phase: the DASPERSE study
Source: Ann Hematol. 2018 Mar 20;97(8):1357–67. doi: 10.1007/s00277-018-3295-8 (PMC6018625; doi:10.1007/s00277-018-3295-8)

**Online Resource**

**Online Resource Methods**

A repeated logistic regression mixed model was used for safety analyses to account for within-patient correlations (e.g., a patient with improvement of one adverse event [AE] is probably more likely to have improvement of another AE) to generate accurate standard errors and 95% confidence intervals (CIs). Efficacy analyses included 95% CIs for the proportion of patients with major molecular response (MMR) and deep molecular response (MR^4.5^) at 6 and 12 months who also had improvement of at least one AE. Descriptive statistics for changes in all patient-reported outcome assessments from baseline will be reported at defined time points (Online Resource Table 1S). Clinically important differences in MD Anderson Symptom Inventory (MDASI) scores were identified by comparing score changes to the minimum important difference (MID).^21^ The MID for MDASI scoring was set to half a standard deviation from the assessment score at patient screening.^22^

**Online Resource Table 1S** Efficacy and outcomes assessment testing schedule

| Time of assessment | | Physical exam | AE assessment | Hematology^a^ | Echo | ECG | Chest  x-ray | BM cytogenetics^b^ | qPCR | MDASI-CML | EORTC QoL/WPAI |
| --- | --- | --- | --- | --- | --- | --- | --- | --- | --- | --- | --- |
| Baseline | | ● | ● | ● | ● | ● | ● | ● | ● | ● | ● |
| Week | 2 |  | ● | ● |  |  |  |  |  | ● |  |
|  | 4 | ● | ● | ● |  |  |  |  | ● | ● |  |
|  | 8 | ● | ● | ● |  |  |  |  | ● | ● | ● |
| Month | 3 | ● | ● | ● |  |  |  |  | ● | ● | ● |
|  | 6 | ● | ● | ● |  |  | ● | ● | ● | ● | ● |
|  | 9 | ● | ● | ● |  |  |  |  | ● |  |  |
|  | 12 | ● | ● | ● | ● |  |  | ● | ● | ● | ● |

*AE* adverse event, *BM* bone marrow, *ECG* electrocardiogram, *Echo* echocardiogram, *EORTC* European Organisation for Research and Treatment of Cancer, *MDASI-CML* MD Anderson Symptom Inventory for CML, *QoL* quality of life, *qPCR* quantitative polymerase chain reaction, *WPAI* Work Productivity Activity Impairment

^a^Complete blood panel with differential, platelets

^b^Bone marrow aspirate is only required at months 6 and 12 if the patients had not already achieved CCyR or MMR

**Online Resource Fig. 1S**  Percentage improvement of imatinib-related adverse events (AEs) within 3 months of switching to dasatinib in all treated patients. Imatinib-related AEs that had ≥70% improvement 3 months after patients switched to dasatinib are displayed, along with 95% confidence intervals (CIs)


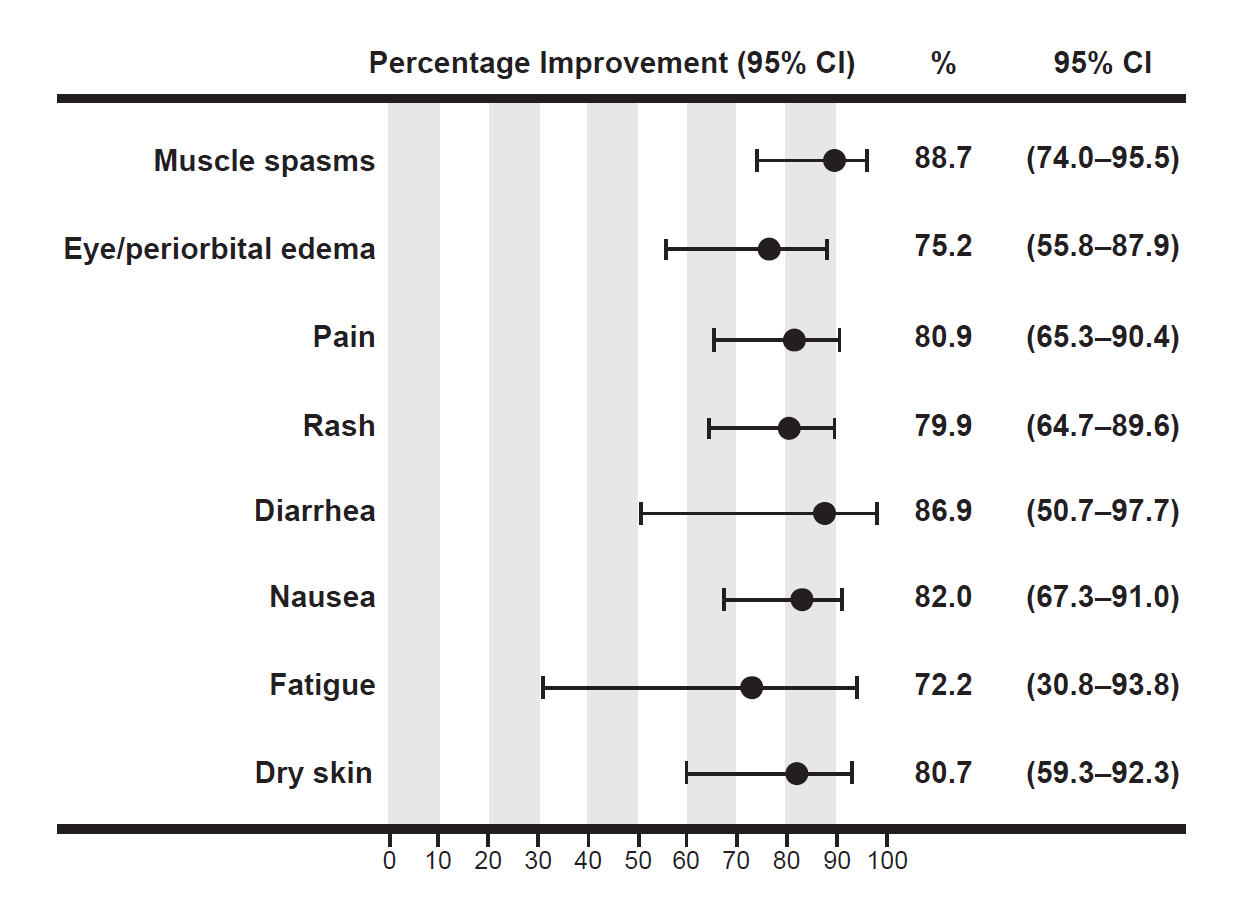

Supplement: Supplementary file 1 — (DOCX 107 kb) [file 277_2018_3295_MOESM1_ESM.docx]
